# Implication of Lipids in Calcified Aortic Valve Pathogenesis: Why Did Statins Fail?

**DOI:** 10.3390/jcm11123331

**Published:** 2022-06-10

**Authors:** Mohamed J. Nsaibia, Anichavezhi Devendran, Eshak Goubaa, Jamal Bouitbir, Romain Capoulade, Rihab Bouchareb

**Affiliations:** 1 Department of Cell Biology and Molecular Medicine, Rutgers University, Newark, NJ 07103, USA; medscience82@gmail.com; 2 Department of Medicine, Cardiovascular Research Institute, The Icahn School of Medicine at Mount Sinai, New York, NY 10029, USA; anichavezhi.devendran@mssm.edu; 3 Thomas Jefferson University East Falls, Philadelphia, PA 19144, USA; shkgoubaa@gmail.com; 4 Department of Pharmaceutical Sciences, Division of Molecular and Systems Toxicology, University of Basel, 4056 Basel, Switzerland; jamal.bouitbir@unibas.ch; 5 L’institut Du Thorax, Nantes Université, CNRS, INSERM, F-44000 Nantes, France; romain.capoulade@univ-nantes.fr; 6 Department of Medicine, Division of Nephrology, The Icahn School of Medicine at Mount Sinai, New York, NY 10029, USA

**Keywords:** aortic valve, lipids, statins, Lp(a), PCSK9

## Abstract

Calcific Aortic Valve Disease (CAVD) is a fibrocalcific disease. Lipoproteins and oxidized phospholipids play a substantial role in CAVD; the level of Lp(a) has been shown to accelerate the progression of valve calcification. Indeed, oxidized phospholipids carried by Lp(a) into the aortic valve stimulate endothelial dysfunction and promote inflammation. Inflammation and growth factors actively promote the synthesis of the extracellular matrix (ECM) and trigger an osteogenic program. The accumulation of ECM proteins promotes lipid adhesion to valve tissue, which could initiate the osteogenic program in interstitial valve cells. Statin treatment has been shown to have the ability to diminish the death rate in subjects with atherosclerotic impediments by decreasing the serum LDL cholesterol levels. However, the use of HMG-CoA inhibitors (statins) as cholesterol-lowering therapy did not significantly reduce the progression or the severity of aortic valve calcification. However, new clinical trials targeting Lp(a) or PCSK9 are showing promising results in reducing the severity of aortic stenosis. In this review, we discuss the implication of lipids in aortic valve calcification and the current findings on the effect of lipid-lowering therapy in aortic stenosis.

## 1. Introduction—Calcific Aortic Valve Disease (CAVD)

Calcific Aortic Valve Disease (CAVD) is the most prevalent heart valve disorder in developed countries; it is a progressive fibrocalcific disease [1,2]. Different risk factors, such as age, male gender, hypertension, metabolic syndrome, diabetes, and bicuspid aortic valve (BAV), have been associated with CAVD [1]. The pathophysiology of CAVD is complex, involving several pathological processes including lipid retention, oxidation, chronic inflammation, fibrotic remodeling, and calcification. Histological analysis of explanted calcified aortic valves revealed the presence of osteoblast-like cells, chondrocytes [3], and inflammatory cells [4,5]. Inflammation and growth factors play an important role in promoting extracellular matrix (ECM) synthesis and in triggering the activation of the osteogenic program in valvular interstitial cells (VICs) [6,7,8,9]. Indeed, proteomics analysis of explanted human pathological aortic valves showed significant disruption of ECM components [7]. Proteoglycans, such as biglycan and decorin, play a major role in lipid retention and modification in CAVD [8,10,11]. In this regard, biglycan, which is an endogenous agonist of Toll-like receptor 2 (TLR2), has been shown to promote inflammation and the osteogenic transdifferentiation of VICs [11].

Moreover, genome-wide association and Mendelian randomization studies have pointed out the association of low-density lipoproteins (LDLs) and lipoprotein(a) (Lp(a)) with the development of CAVD [12,13]. In this regard, several mechanistic studies have confirmed the implication of Lp(a) and its lipid content in the activation of the osteogenic program of VICs [14,15,16].

Although lipids seem to be a key factor in the pathogenesis of CAVD, three randomized clinical trials (RCTs) failed to demonstrate any significant benefit of LDL lowering with statins on the progression of aortic stenosis [17,18,19]. Furthermore, plasma levels of Lp(a) are not significantly modified by statin therapy [20]. Therefore, there is significant interest in targeting Lp(a) levels with novel therapeutic agents, such as antisense oligonucleotides, to slow the progression of CAVD [21,22,23]. Furthermore, Langsted et al. (2016) found that patients with a PCSK9 loss-of-function mutation (PCSK9 R46L) have lower serum LDL and Lp(a) levels and a lower risk of calcific aortic stenosis based on data from 103,083 people in the Copenhagen General Population Study [24]. Herein, we examine the implication of lipids in CAVD. In addition, we have placed special emphasis on statins’ failure to reduce the progression of aortic stenosis.

## 2. Role of Lipoprotein(a) and Oxidized Phospholipids in CAVD

### 2.1. The Implication of Nitric Oxide (NO) Activity in CAVD

Endothelial nitric oxide synthase (eNOS) uncoupling appears to be one central mechanism during early-stage disease and contributes to CAVD progression [25]. Indeed, NO synthesis and signaling are markedly affected by the oscillatory shear stress in the endothelium lining the calcification-prone fibrosa compared with the disease-resilient ventricularis [25]. In vivo studies have shown that a lack of eNOS in mice promotes a CAVD-like phenotype [26], whereas restoring paracrine NO signaling blunts VIC-driven calcification in different experimental models [27]. Furthermore, studies have also reported that NO maintains valvular homeostasis through guanylyl cyclase/cGMP- and NOTCH1-dependent mechanisms [28,29]. More recently, Majumdar et al. (2021) deepened mechanistic insights by showing that valvular endothelial cell (VEC)-derived NO rescues calcification by an S-nitrosylation-mediated mechanism in porcine aortic valve interstitial cells. The alteration of nitric oxide (NO) signaling and activity in CAVD induces reactive oxygen species generation, which may promote the production of oxidative lipid species, triggering inflammation signaling activation in the aortic valve [27]. Targeting eNOS may open new therapeutic avenues to restore the paracrine homeostasis of endothelial/valve interstitial cells [29].

### 2.2. The LPA Gene Locus and CAVD

The *LPA* gene locus determines circulating Lp(a) levels primarily, with no significant dietary or environmental influences (Thanassoulis et al., 2013). The *LPA* gene is located on chromosome 6 (6q25.3-q26) and has a high degree of homology with the plasminogen gene (PLG). The *LPA* locus is complex, with copy number variants (CNVs) in the region encoding the kringle IV type 2 (KIV2) domain, which is inversely related to Lp(a) levels in the blood [30]. Genome-wide association (GWA) studies and Mendelian randomization (MR) analyses have underlined a causal association between a common gene variant, rs10455872, located in the *LPA* gene locus and CAVD [12,31].

However, a recent GWA study did not find an association between *LPA* and CAVD in patients with a congenital bicuspid aortic valve [31]. A large meta-analysis conducted on 1797 CAVD cases and 131,932 controls revealed that carriers of rs10455872 have a 1.66-fold higher risk of developing CAVD [32]. Furthermore, studies showed that genetically determined lower levels of Lp(a) were associated with a 37% reduced risk of CAVD [33]. These findings suggest that lowering Lp(a) levels and/or blocking the specific pathways by which Lp(a) promotes CAVD could lead to therapies that slow the progression of CAVD.

### 2.3. Lp(a), a Major Carrier of Oxidized Phospholipids (Ox-PL), Is a Risk Factor for Aortic Stenosis

Lp(a) is composed of low-density lipoprotein (LDL)-like particles in which apolipoproteinB-100 (apoB) is covalently bound by a single disulfide bond to the glycoprotein apolipoprotein(a) (apo(a)) [30,34]. Apolipoprotein(a) is highly polymorphic, with a variable number of (KIV2) domains. The copy number variant of KIV2 domains determines the length of the lipoprotein, and it is inversely related to the level of Lp(a) in circulation. Studies have underlined the high-content oxidized phospholipids (Ox-PL) in Lp(a) particles [35]. Ox-PL binds to the KIV type 10 domain and is thus ferried, as a cargo, by Lp(a). Hence, Ox-PLs carried by Lp(a) may contribute to endothelial dysfunction, inflammation, and the expression of genes with pro-calcifying properties [35].

Ox-PL has been established as a causal risk factor for AS in several genetic and population studies [12,31,36]. The genetically determined level of Ox-PL linked to apo(a) (Ox-PL-apo(a)) increases the risk of CAVD 1.09-fold [37]. Interestingly, clinical imaging with 18F sodium fluoride positron emission tomography or computed tomography has revealed the presence of aortic valve micro-calcification in individuals with elevated Lp(a) prior to the development of clinical manifestations of CAVD (Despres et al., 2019). Together, these studies thus highlight that Lp(a) and its cargo, Ox-PL, are involved in the development of CAVD.

### 2.4. Lipid Oxidation Promotes Calcific Aortic Stenosis

Increasing evidence suggests that the infiltration of lipoproteins into the aortic valve plays a central role in promoting inflammation, which, in turn, might induce the activation of the osteogenic program in VICs [14,38]. Histological analysis of explanted calcified aortic valves has revealed the presence of several apolipoproteins (apo), such as apoB, apoE, apoA1, apolipoprotein E, and apo(a) [39,40,41]. Furthermore, there is an association between the level of Ox-LDL, the degree of inflammation, and fibrocalcific remodeling [42,43].

**Figure 1:**
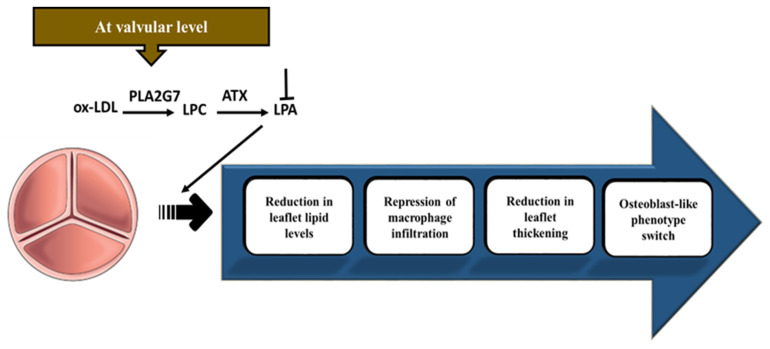
The implication of Ox-LDL in the calcification of the aortic valve. The infiltration of Ox-LDL into the aortic valve activates inflammation and, consequently, the release of PLA2G7 from macrophages, leading to the production of LysoPC. The activation of autotaxin leads to the preproduction of LPA, which amplifies the inflammation and the activation of the osteoblastic-like phenotype switch.

In vitro studies showed that Ox-LDL and several oxidized phospholipid (Ox-PL) species carried by the Lp(a) fraction promote the calcification of VICs [44]. In turn, lipoprotein-associated phospholipase A2 (Lp-PLA2) transforms Ox-PLs into lysophosphatidylcholine (LysoPC), which acts as a reactive metabolite that promotes the mineralization of VICs [45]. Immunohistochemical studies have highlighted the co-localization of Lp-PLA2 with Ox-LDL, suggesting that Lp-PLA2 could be transported by lipoproteins into the aortic valve [45,46,47]. Together, these studies suggest that the accumulation of oxidized lipids triggers osteogenic response activation in VICs [35] (Figure 1).

## 3. Autotaxin (ATX)–Lysophosphatidic Acid (LysoPA) Axis Mediates Mineralization of the Aortic Valve

Autotaxin (ATX) is a member of the ecto-nucleotidase family of enzymes encoded by the ENPP2 gene [48]. It was initially isolated from melanoma cell lines and was identified as a motility factor [49]. ATX is a secreted glycoprotein that hydrolyzes lysophosphatidylcholine (LysoPC) into lysophosphatidic acid (LysoPA). LysoPA is an active metabolite with potent and diverse biological properties. It promotes cell motility, inflammation, calcification, and fibrosis [49]. It is believed that the majority of circulating LysoPA is derived from ATX [50]. According to Bouchareb et al. (2015), ATX is likely transported into the aortic valve by Lp(a), and it is also secreted by VICs in response to inflammatory stimuli [15]. Indeed, ATX activity is enriched in an isolated fraction of Lp(a). Moreover, binding assay analysis, using human purified Lp(a), confirmed the physical association between Lp(a) and ATX [51]. In vitro inhibition of ATX prevented the mineralization of VICs induced by LysoPC, suggesting that LysoPA is the mediator promoting the activation of the osteogenic program in VICs [15]. Of interest, ATX expression and activity were increased in human explanted pathological aortic valves [15]. To this effect, stimulation of VICs with LysoPC and Ox-PLs treatment induces the expression of ATX [15,38]. Moreover, the administration of LysoPA to LDR^-/-^ apoB^100/100^ IGFII mice increased the osteogenic activity in the aortic valve and accelerated the development of CAVD [15,38]. Following a series of in-depth investigations, it has been shown that ATX and LysoPA promote aortic valve inflammation and mineralization through the activation of the NF-κB/bone morphogenetic 2 (BMP2) pathway [15,38]. In this regard, a significant interaction term was found between ATX activity and Lp(a) level [16]. Together, these studies indicate that ATX is carried by Lp(a) and is also secreted by VICs, increasing LysoPA levels and, therefore, stimulating inflammation.

More recently, Bouchareb et al. (2019) also showed that activated platelets promote VIC mineralization in vitro through the activation of ATX. In addition, ATX activity was higher in platelets from patients with CAVD compared to control patients (Bouchareb et al., 2019).

ATX promotes inflammation and the osteogenic transdifferentiation of VICs through the production of LysoPA, which is a small lipid derivative acting on G-protein coupled receptors with various biological functions [38]. In vitro studies have shown that oxidized LDL (Ox-LDL) induces the mineralization of VIC cultures, whereas treatment with an antagonist of LPAR1 prevents this effect [38]. The same study has also highlighted the overexpression of LPAR1 in human calcified aortic valves. In vitro studies using human VICs showed that lysoPA treatment stimulates the expression of the bone morphogenetic protein (BMP2) via the activation of the NFkB pathway. The promoter region of BMP2 contains NFkB-responsive elements and LysoPA promotes the phosphorylation of p65 on serine 536 (p65 S536). Of particular interest, phosphorylated p65 S536 was recruited to the promoter of BMP2 to activate BMP2 gene expression [38]. The pharmacological inhibition of LPAR1 with Ki16425 in LDLR^-/-^ apoB ^100/100^ IGFII mice reduced the progression of CAVD and downregulated the expression of BMP2 in aortic valve cusps [38].

Lastly, Mkannez et al. (2018) [52] have recently underlined that the expression and the enzymatic activity of PLPP3 (also known as PPAP2B), a phospholipid phosphatase that inactivates LysoPA, were decreased in human calcified aortic valves compared to controls. Consistently, aortic valves with lower expression of PLPP3 had an increased level of LysoPA [52]. Furthermore, the knockdown of PLPP3 exacerbated the LysoPA-induced expression of BMP2 and consequently simulated in vitro VICs’ mineralization [52].

## 4. The Implication of apoC-III in the Calcification of the Aortic Valve

Metabolic syndrome is described as a dysmetabolism related to insulin resistance and visceral obesity, which leads to a pro-inflammatory and pro-thrombotic state [53]. This syndrome has been associated with the increased incidence and progression of aortic valve calcification [54] and hemodynamic progression of aortic valve stenosis [55], making the visceral obesity-related perturbations a potential target to reduce the development and progression of CAVD.

One of the main features of metabolic syndrome is hypertriglyceridemia. The apolipoprotein C-III (apoC-III) is associated with elevated triglyceride levels. As opposed to other apolipoproteins, such as apo(a) or apoB, multiple particles of ApoC-III are carried by all lipoproteins [56,57]. This finding supports the use of ApoC-III as a potential biomarker, independent of the other lipid factors, and a potential therapeutic target. Indeed, ApoC-III’s circulating levels have been linked to an increased risk of cardiovascular events, and targeting this apolipoprotein is a promising way to lower the risk for these patients [58,59,60,61].

ApoC-III is described as a multifunctional protein, playing a role in the metabolism of several lipoproteins, glucose homeostasis, endothelial cell dysfunction, inflammation, and the coagulation cascade, and in increasing lipoprotein affinity to the extracellular matrix (especially proteoglycans) [57]. Interestingly, all these features have also been associated with the development and/or progression of CAVD and support a potential detrimental action of this apolipoprotein in the pathogenesis of CAVD. This was further studied in a post-hoc analysis of the ASTRONOMER trial, where the Lp(a) content in ApoC-III has been related to faster CAVD progression [61]. As previously stated, this study provides evidence that, in addition to Lp(a) plasma levels, the content of this lipoprotein would be of great interest in understanding the mechanisms leading to aortic valve calcification and the development of aortic stenosis. Further studies focused on these aspects are needed and, by compiling data, would provide mechanistic evidence to target these lipoproteins in the context of CAVD.

## 5. The Effect of Lipid-Lowering Therapy in Aortic Stenosis

### 5.1. Rationale of Statins

Statins or the hydroxymethylglutaryl-CoA reductase (HMG-CoA) inhibitors are considered a potent therapeutic strategy in patients with atherosclerotic plaques. Statins are unquestionably well documented for their lipid-lowering effects [62,63]. Indeed, statins have demonstrated an improved survival rate in subjects with atherosclerotic coronary heart disease (CHD) [23]. They target the HMG-CoA reductase that catalyzes the switching of HMG-CoA to mevalonic acid, which abolishes the production of cholesterol [64]. The inhibition of HMG-CoA reduces cholesterol levels through the up-regulation of hepatocyte-LDL receptors, thus increasing the uptake of circulating LDL-cholesterol into the hepatocytes and subsequently decreasing LDL levels [65].

### 5.2. Statins’ Mechanism of Action

**Figure 2:**
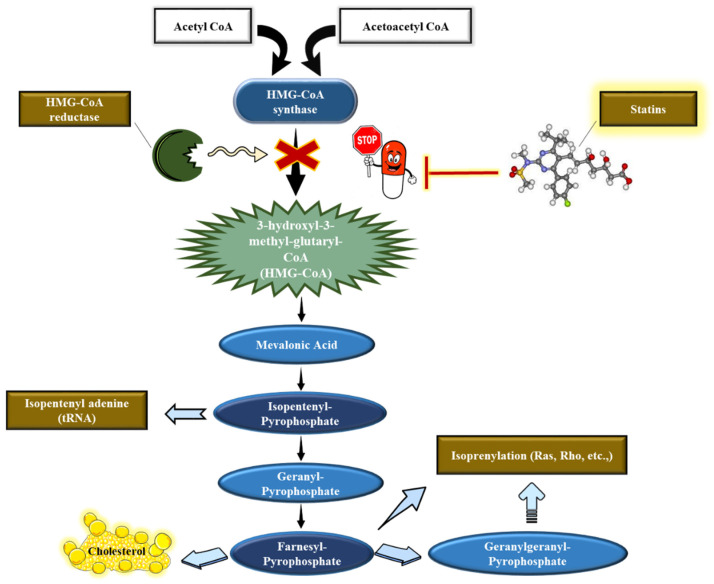
Statins’ mechanism of action. Statins inhibit the HMG-CoA reductase to block the synthesis of mevalonic acid and, consequently, the production of cholesterol.

**Figure 3:**
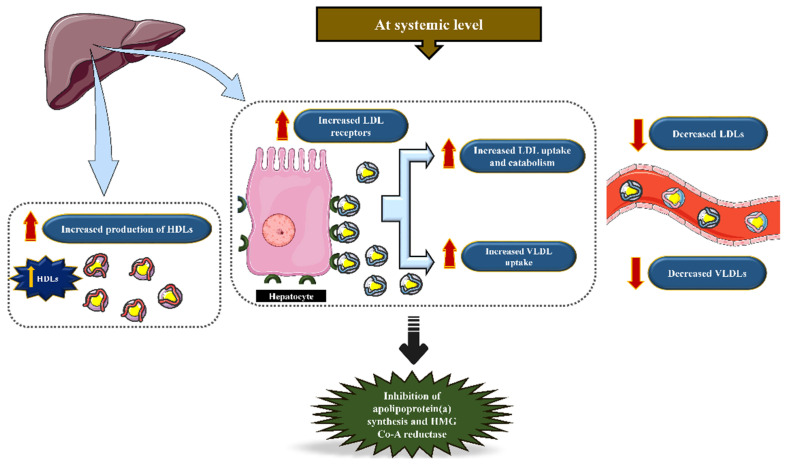
The effects of statins at systemic levels. Statins increase the expression of LDL receptors on hepatocytes to increase LDL uptake and catabolism, leading to a systemic decrease in LDLs, vLDLs, and the production of HDL.

Statins obstruct HMG-CoA reductases, the pivotal catalytic enzyme in the cholesterol biosynthesis pathway (Figure 2 and Figure 3). This enzyme predominantly regulates the conversion of HMG-CoA synthases to mevalonic acid and, hence, in the course of action, manifests a decline in the plasma LDL levels [62]. Primarily, the lipid-lowering pleiotropic effects of statins are supposedly based on hampering the production of significant isoprenoid intermediates such as farnesyl pyrophosphate and geranylgeranyl pyrophosphate in the cholesterol biosynthesis pathway [66,67,68,69]. In addition, they were proven to block the destabilizing effects of mevalonate on nitric oxide synthase (NOS)-mRNA in human endothelial cells (ECs), thereby resulting in the increased synthesis and function of the NOS enzyme [23].

### 5.3. Statin-Mediated Lipid-Lowering Therapeutic Approaches to Target CAVD: Past, Present, and Future Prospects

The existence of similarities in the pathophysiology of atherosclerosis and aortic stenosis has led the clinical and research community to consider statins as a treatment to decelerate the progression or to reduce the severity of aortic stenosis. Indeed, studies are showing the implication of Ox-LDL in the activation of osteogenic transition in calcified aortic valves [5,16,38,45]. In light of the facts presented, these results elicited considerable interest among the scientific community that eventually paved the way to the initiation of numerous randomized controlled clinical trials to elucidate the effect of a lipid-lowering therapeutic regimen in aortic stenosis. Clinical studies, however, found no benefit of statins in terms of the hemodynamic progression or disease severity of aortic stenosis [17,18]. In the Simvastatin and Ezetimibe in Aortic Stenosis (SEAS) trial, patients were randomized to receive simvastatin 40 mg plus ezetimibe 10 mg, or a placebo [18,70]. The drug combination significantly lowered LDL levels compared to the placebo. However, no effect on the progression of aortic stenosis was observed [70]. To further complicate the situation, another meta-analysis study by Teo et al. [71] using randomized placebo-controlled clinical trials on 2344 patients reported no differences in clinical outcomes between the placebo and the treatment group. As a result of these negative outcomes of the conducted trials, the American Heart Association/American College of Cardiology and the European Society of Cardiology guidelines together do not endorse the use of statins for the treatment of CAVD [72,73].

**Figure 4:**
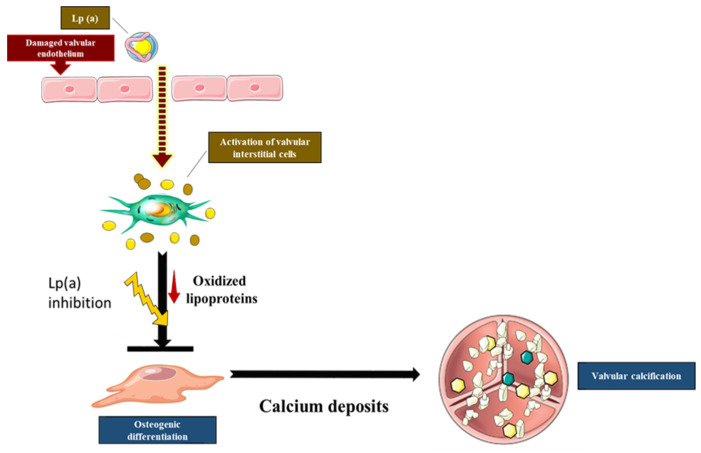
The effect of Lp(a) on aortic valve calcification. Lp(a) particles carry oxidized LDL, which stimulate aortic valve cells’ calcification. Targeting Lp(a) might inhibit valve inflammation and, consequently, reduce valve cell calcification.

Nonetheless, the Aortic Stenosis Progression Observation: Measuring Effects of Rosuvastatin (ASTRONOMER) study showed an association between elevated levels of oxidized phospholipids and lipoprotein(a) (Lp(a)) in patients with accelerated hemodynamic progression of CAVD [74]. These findings support the hypothesis that Lp(a) mediates AS progression through its binding to Ox-PL [15,37,74]. These findings paved the way to the implementation of randomized clinical trials focusing on Lp(a)-lowering therapy in mild-to-moderate CAVS patients with elevated Lp(a) plasma levels (Figure 4).

Two randomized, double-blind, placebo-controlled trials are ongoing. In patients with elevated Lp(a), phase 1 and 2 trials demonstrated the tolerability, safety, and beneficial effect of lowering Lp(a) concentrations with IONIS-APO(a)Rx, an oligonucleotide targeting apolipoprotein(a). This therapeutic agent was found to reduce circulating Lp(a) by 80% (Koren et al., 2022; Tsimikas et al., 2015; Viney et al., 2016). A phase 3 clinical trial (Lp(a) HORIZON trial; NCT04023552) is now underway to assess the impact of this treatment on clinical outcomes in patients with established cardiovascular disease and elevated Lp(a) plasma levels.

Proprotein convertase subtilisin/Kexin type 9 (PCSK9), an enzyme that is formed in the liver, has been described as a central player in cholesterol metabolism, particularly because PCSK9 stimulates the degradation of the LDL receptor and then leads to an increase in circulating LDL [75]. Inhibition of PCSK9 and its associated drastic reduction in circulating LDL suggests a promising treatment for patients with cardiovascular diseases [76,77]. In addition to lowering LDL, PCSK9 inhibitors were associated with a 15 to 30% reduction in Lp(a) plasma levels, which may benefit CAVD patients. Indeed, Langsted et al. [24] have shown, in 103,083 individuals from the Copenhagen General Population Study, that patients with loss-of-function mutation of PCSK9 (PCSK9 R46L) are associated with decreased serum LDL and Lp(a) levels and a reduced risk of calcific aortic stenosis (CAVD). With this underlying substantial evidence, the PCSK9 inhibitors (deliberated as monoclonal antibodies: alirocumab and evolocumab) are currently being examined as pharmacological routes to delay the progression of aortic stenosis [78]. The exploratory investigations from the Further Cardiovascular Outcomes Research with PCSK9 Inhibition in Subjects with Elevated Risk (FOURIER) randomized clinical trial support this approach. In this post-hoc analysis, patients randomized to evolocumab had a 50 % decrease in the incidence of CAVS over a median follow-up of 2.2 (1.8–2.5) years [79]. Furthermore, recent experimental studies [75,80] have shown that PCSK9 could be involved in the process leading to aortic valve calcification and that the in vitro inhibition of PCSK9 could decrease VIC calcification.

### 5.4. Statins’ Off-Target Effects

**Figure 5:**
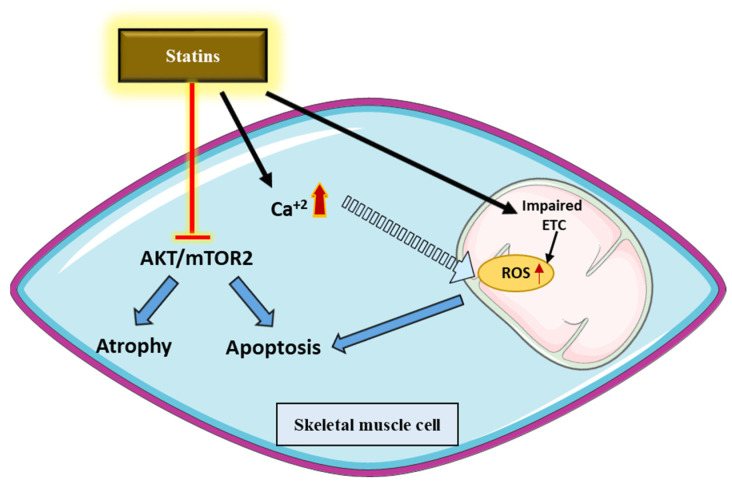
Off-target effects of statins. Statins act at different levels in skeletal muscle cells. First, they provoke the increased release of calcium from the sarcoplasmic reticulum to the cytoplasm and perturb the contractility of muscle fibers. In parallel, statins impair mitochondrial function, leading to the accumulation of ROS and to the activation of apoptosis. Finally, statins impair the function of Akt due to the impaired insulin signaling pathway and due to the impaired function of mTORC2. As a result, statins induce increased protein degradation and impaired protein synthesis, promoting skeletal muscle atrophy [95].

Increased evidence points out several off-target effects of statins (Ward et al., 2019), including statin-associated muscle symptoms (SAMS), diabetes mellitus (DM), and effects on the central nervous system (Thompson et al., 2016) (Figure 5).

One of the main overlooked aspects in the context of statins’ myotoxicity is calcium signaling. Studies showed that only lipophilic statins stimulate calcium release from the sarcoplasmic reticulum in rat and human skeletal muscle [81,82]. Acute applications of simvastatin on skeletal muscle fibers increased the cytosolic Ca^2+^ concentrations released from the sarcoplasmic reticulum [83]. Moreover, a previous study showed that simvastatin impaired ryanodine receptor 1 (RyR1) Ca^2+^ function, causing aberrant Ca^2+^ handling, which led ultimately to cell apoptosis [84].

The mevalonate pathway (Figure 2) produces ubiquinone or coenzyme Q10, which is an important player in the mitochondrial electron transport chain (ETC). Studies by Bouitbir and others showed clearly that statins impair mitochondrial function at different levels (Figure 5) [85,86,87,88]. For instance, simvastatin inhibits complex I activity, as observed in rat and human skeletal muscles treated with simvastatin [82]. The inhibition of mitochondrial function was only observed with lipophilic statins and was not rescued by the addition of cholesterol intermediate mevalonate, which would indicate that statins directly impair mitochondrial function, independently of the inhibition of HMG-CoA reductase [88]. Furthermore, patients treated with statins and experiencing muscle-related side effects presented impaired mitochondrial function, lower mitochondrial content, and increased mitochondrial reactive oxygen (ROS) production in deltoid biopsies [86]. However, the same study showed that statins increased mitochondrial function in heart biopsies of the same patients [86]. These findings showed clearly that oxidative muscles such as the heart are more resistant to statins than glycolytic muscles, most likely due to the higher mitochondrial content and higher anti-oxidative capacities (Figure 5).

Akt represents an important kinase governing the homeostasis between cell growth, survival, and metabolism. Several in vitro studies documented an impaired Akt function and its downstream signaling pathway, which lead to cell apoptosis and protein degradation [85,89]. Moreover, it was shown that decreased Akt phosphorylation and impaired mitochondria respiration were responsible for simvastatin-induced myopathy in C2C12 myo-tubes [90].

Recently, new-onset diabetes mellitus has been reported as a new adverse event in patients treated with statins [91]. The incidence of diabetes associated with statin therapy is estimated at up to 30% [92]. The JUPITER trial reported a significant increase in type 2 diabetes in patients treated with rosuvastatin [91]. Moreover, decreased insulin sensitivity and hyperglycemia in hypercholesteremic patients have been observed with atorvastatin [21]. However, the impairment of insulin sensitivity was observed only with lipophilic statins [21]. The impaired insulin signaling and the disturbed GLUT4 synthesis or translocation seem to establish the link between statins and insulin resistance [93]. Moreover, impaired translocation of the GLUT4 vesicles to the cell membrane was observed when cells were exposed to atorvastatin [94]. Statins impair the isoprenylation of several proteins and GTPases by inhibiting cholesterol biosynthesis. Interestingly, this effect was not observed with pravastatin, highlighting a class effect. Because of these various off-target effects and the complex physiopathology of aortic stenosis, statins did not show any beneficial effect on stopping the progression of aortic stenosis.

Finally, the concept of a lipid-lowering therapeutic regimen in CAVS is reassuring. The heterogeneity of lipid species in the aortic valve might explain the failure of statins to reduce the progression of aortic stenosis. However, the clinical trials using antisense oligonucleotides to inhibit the expression of Lp(a) (Figure 4) or the use of monoclonal antibodies to inhibit the expression of PCSK9 still support the implication of lipids in Calcified Aortic Valve Disease. The use of lipidomic studies to explore the circulating and valvular lipid species in patients with CAVS might provide molecular cues for more efficient and compelling therapeutic targets to reduce the progression of aortic stenosis.

## Data Availability

Not applicable.
